# Validation of the oral health impact profile - 14 in patients with head and neck cancer

**DOI:** 10.4317/medoral.23765

**Published:** 2020-05-10

**Authors:** Milan Tesic, Milos Cankovic, Marija Jevtic, Dejan Stevanovic

**Affiliations:** 1 Clinical Hospital Centre Zemun, Department of ENT with maxillofacial surgery, Belgrade, Serbia; 2 Military Medical Academy, Clinic for Maxillofacial surgery, Belgrade, Serbia; 3 University of Novi Sad, Faculty of Medicine; 4 Dentistry Department, Oral Medicine Section, Novi Sad, Serbia; 5 Institute of Public Health Vojvodina, Novi Sad, Serbia; 6 Clinic for neurology and psychiatry for children and youth Belgrade, Serbia

## Abstract

**Background:**

The Oral Health Impact Profile-14 (OHIP-14) was designed to assess patients’ perception of the impact of oral disorders on their quality of life (QoL). Although the OHIP-14 is now frequently used in patients with head and neck cancer, data related to its measurement properties in this population are scarce. The aim of the present study was to evaluate the reliability and validity of the OHIP-14 in a sample of Serbian patients with head and neck cancer.

**Material and Methods:**

Data were available for 345 patients (257 [74.5%] males; aged 30-92 years), with head and neck cancer. All patients completed the OHIP-14 and the European Organization for Research and Treatment of Cancer (EORTC) QLQ-C30 and QLQ-H&N35. Factor analyses, internal consistency reliability (i.e., Cronbach’s α coefficient), and construct validity were analyzed.

**Results:**

The factor analyses confirmed that 14 OHIP items were measuring a single underlying factor. Cronbach’s α coefficient was 0.98 and corrected item-total correlations ranged 0.77-0.93. Lower OHIP-14 scores (i.e., lower impacts on oral health) were more frequently present among patients who had only surgery as a therapeutic procedure compared to those who had surgery accompanied with radio- and chemotherapy (*p*< 0.01). Patients with a tumor stage 0-II also had lower OHIP-14 scores compared to those who had a tumor stage III-IV (*p*< 0.01). The OHIP-14 correlated significantly with the QLQ-C30 and QLQ-H&N35 scales.

**Conclusions:**

As a unidimensional instrument, the OHIP-14 provides oral QoL assessments with sound internal consistency reliability and construct validity among patients with head and neck cancer.

** Key words:**Quality of life, oral health, psychometrics, reliability, validity.

## Introduction

Over the past 30 years, patient reported outcome (PRO) measures have become standard methods for providing additional information on how patients perceive the impacts of their health condition and its treatment on everyday living (1). Especially, significant research attention has been placed to develop PROs that would assess comprehensively survival and living of people with cancer, such as symptoms and side effects, limitations, well-being, functioning, health-related quality of life (QoL).

Head and neck cancer (HNC) affect different locations of the head and neck (2), with physical (e.g., appearance changes or pain) and functional alternations (e.g., changes in mastication, deglutition or phonation), but also multiple psychosocial consequences (e.g., anxiety, depression or social functioning) (2-5). Antineoplastic treatments such as surgery, chemo- and radiotherapy usually result in multiple adverse effects and squeals, too (5). Thus, measuring the impacts of their health condition and its treatment via PROs is now used in many research studies, clinical trials, and clinical settings in patients with HNC.

The oral health of patients with HNC can significantly deteriorate due to the conduction itself and antineoplastic treatments, including mentioned physical conditions and functional changes (6). Oral QoL, as an internationally accepted concept to assess how patients are affected by oral conditions and how they experience the effect of prescribed interventions (7), has been recognized as an important PRO among patients with HNC, too. Among the first, Shavi *et al*. (8) demonstrated a high impact of the oral health on the QoL of patients with HNC, which was confirmed in later studies (6,9-11). In particular, studies showed that patients with HNC who had been treated surgically alone had better QoL compared to the combined treatment modalities (10) and the oral health condition of these patients deteriorates after radiotherapy, with direct impacts on their QoL (6).

The Oral Health Impact Profile (OHIP) is designed to assess individuals’ perception of the impact of oral disorders on their QoL (12). This is a 49-item instrument assessing dysfunction, discomfort, disability, and handicap associated with oral disorders/conditions and their treatments. A short form, the OHIP-14, was developed to represent the main domains of the original, namely functional limitation, physical pain, psychological discomfort, physical, psychological and social disability, and overall handicap (13), and it is frequently used for oral QoL assessments. Available studies demonstrated sound psychometric properties of the OHIP-14 in different populations including different language versions (14-23). Nevertheless, although the OHIP-14 is more and more used in patients with HNC (6,9-11), data related to its psychometric properties in this population are scarce. Good internal consistency (Cronbach’s α = 0.86) was reported in one study with the Brazilian version (9). Data related to different aspects of validity are missing. Therefore, the aim of the present study was to evaluate the reliability and validity of the OHIP-14 in a sample of Serbian patients with HNC, including the dimensionality, internal consistency reliability, and construct validity.

## Material and Methods

- Participants

This is a cross-sectional, psychometric study that included patients treated for HNC, who were admitted to Department of ENT with maxillofacial surgery of Clinical Hospital Centre Zemun, Belgrade, Serbia. As a sample of convenience, all patients were recruited during pre-scheduled assessments when visiting a doctor for follow-up care. The participation was on a voluntary basis and the only exclusion criterion was inability to read and/or write. To all patients was first explained the aim of the study and all included patients provided written informed consent before participating in the study. The instruments (see below) were self-completed by included patients.

Complete data for analyses were available for 345 patients with HNC (257 [74.5%] males), aged 30-92 years (Table 1).

- Instruments

OHIP-14: The OHIP-14 has 14 items with answers rated on a 5-point Likert scale (from 1 = never to 5 = very often) to indicate a level of different problems related to oral health over in the last 12 months (13). The total score is the sum of all answered items and it can range from 0 to 56. The higher the score, the worse the impact on oral health is. The Serbian version was developed in an earlier study (24), and adapted by the one of the author of this study, and it has the same format and contents as the original.

European Organization for Research and Treatment of Cancer (EORTC) QLQ-C30 and QLQ-H&N35: The QLQ-C30 is a 30-item, cancer-specific instrument for QoL assessments (25). It has the following multi-item function/symptom scales: Global health/QoL, Physical, Role, Emotional, Cognitive, and Social functioning, Fatigue, Nausea and vomiting, Pain, Dyspnea, Insomnia, Appetite loss, Constipation, Diarrhea, and Financial difficulties. As a supplementary module, the QLQ-H&N35 is a 35-item instrument for the assessments of symptoms associated specifically with HNC (26). It has the following symptom scales: Pain in the mouth, Swallowing, Senses, Speech, Trouble with social eating, Trouble with social contact, Sexuality, Problems with teeth, Problems opening mouth, Dry mouth, Sticky saliva, Coughing, Felt ill, Painkillers, Nutritional supplements, Feeding tube, Weight loss, and Weight gain.

**Table 1:**
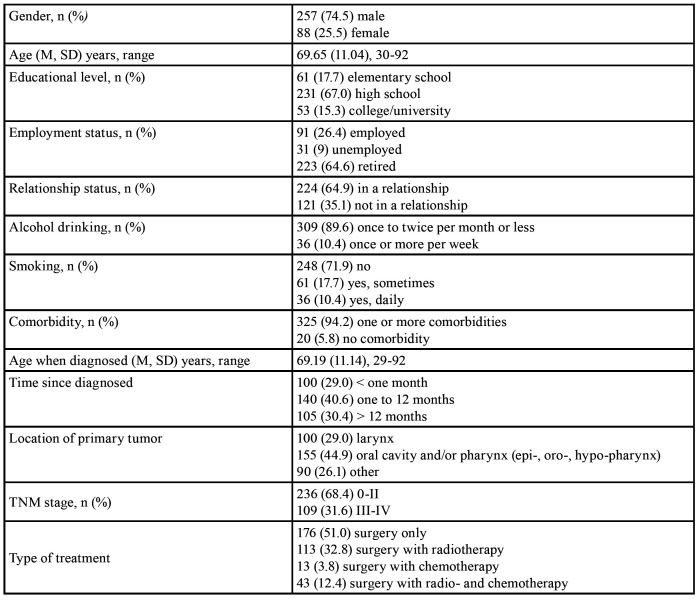
Characteristics of studied patients (n = 345).

Scores for all scales of the both instruments are calculated by linear transformation of raw scores into a 0–100 score, where scores of 100 represent the best outcomes on the QLQ-C30 functioning scales and the worst outcomes on the QLQ-C30 and QLQ-H&N35 scales. The Serbian versions were provided by the EORTC Group.

- Statistical analysis

Statistical analyses included the assessment of descriptive characteristics, factor structure (i.e., exploratory factor analysis [EFA] and confirmatory factor analysis [CFA]), internal consistency reliability, and construct validity (i.e., know-group and convergent/discriminant validity). To carry out the analyses, SPSS Statistics Version 24 and AMOS Version 18 were used.

Factor structure: To explore the factor structure (i.e., EFA and CFA), the whole sample was randomly split. Principal axis factoring (PAF) with Promax (oblique) extraction method, as an EFA method, was used with the data of 162 participants (one half of the sample). Afterwards, 14 items were subjected to a maximum likelihood solution with the second sample of 183 participants (the other half) was employed to test CFA. A series mean repletion was used to handle missing data as the amount was negligible per item (0.3–0.9%). Absolute model fit to the data for the CFA was evaluated using the comparative fit index (CFI), Tucker-Lewis index (TLI), and root mean square error of approximation (RMSEA) with the following cut-offs (27): TLI and CFI ≥ .90, RMSEA ≤ .08 as adequate; TLI and CFI ≥ .95, RMSEA ≤ .06 as good fit; RMSEA ≤ .10 as marginal fit.

Descriptives and internal consistency reliability: The distribution of mean scores, standard deviation (SD), and percentage floor and ceiling effects were calculated. A floor and ceiling effect were defined as the percentage of individuals with the best and worst results respectfully. More than 15% of participants with the highest or lowest score on one particular scale were considered as a relevant effect. Internal consistency reliability was evaluated using Cronbach’s α coefficient and the values of α ≥ 0.7 were considered accepTable.

Know-group validity: A t-test and one-way analysis of variance (ANOVA) were used to test for differences in OHIP-14 scores among different groups for which it would be expected that OHIP-14 scores differ. It was hypothesized that patients with cancer located other-then-oral regions would have lower scores (i.e., lower impacts on oral health) then those with oral cavity and pharyngeal cancer (epi/oro/hypo pharynx) or laryngeal cancer as well as those patients who had only surgery as a therapeutic procedure compared to those who had surgery accompanied with radio- and chemotherapy patients, and patients with a TNM stage 0-II compared to a TNM stage III-IV.

Convergent/discriminant validity: Convergent and discriminant validity was evaluated with Pearson correlation coefficients between the OHIP-14 and the QLQ-C30 and QLQ-H&N35 scales. Correlation coefficients ranging 0.1–0.3 were considered low, those 0.31–0.5 moderate, and those exceeding 0.5 high.

## Results

- Factor Structure

The appropriateness for conducting the EFA was confirmed by the results of the Kaiser–Meyer–Olkin Measure of Sampling Adequacy (KMO = 0.96) and Bartlett’s Test of Sphericity (χ2 (df) = 3286.54 [91], *p* < 0.01). The PAF analysis revealed a single underlying factor explaining 79.7% of the total variance, with all 14 items having high factor loadings (ranging 0.66-0.95). Afterwards, a CFA analysis of model showed the following fit indexes (χ2 (df) = 285.49 [77]; TLI = 0.93, CFI = 0.94, and RMSEA = 0.12, with all standardized regression weights being statistically significant (ranging 0.63-0.95; *p* < 0.01). On the same sample was also tested the model of seven correlated factors (15,16) with the following fit indexes obtained: χ2 (df) = 174.14 [56]; TLI = 0.94, CFI = 0.97, and RMSEA = 0.11. In addition, the model with three correlated factors proposed by Zucoloto *et al*. (23) was also tested and the following fit indexes were obtained: χ2 (df) = 254.81 [74]; TLI = 0.94, CFI = 0.95, and RMSEA = 0.12.

- Internal consistency reliability

For the total sample Cronbach’s α coefficient was 0.98 and corrected item-total correlations ranged 0.77-0.93. Cronbach’s α coefficient was > 0.92 for five groups of patients (Table 2). The percentages of patients having the highest or lowest OHIP score were < 4 indicating on the absence of floor and ceiling effects.

- Known-group validity

Patients who had only surgery as a therapeutic procedure had significantly lower OHIP-14 scores compared to those patients who had surgery accompanied with radio- and chemotherapy (t (df) = -15.99 [343]; *p* < 0.01). In addition, patients with a TNM stage 0-II had significantly lower OHIP-14 scores compared to those who had a TNM stage III-IV (t (df) = 11.05 [343]; *p* < 0.01). Based on ANOVA, statistically significant differences in the OHIP-14 score were found when comparing patients with three different localizations of cancer (F [2, 342] = 22.63, *p*< 0.01; Table 2). As predicted, patients with laryngeal and with oral cavity and pharyngeal carcinoma had significantly higher scores compared to those with cancer located on the other sites of the head and neck (*p* < 0.01).

- Convergent/Discriminant Validity

Except with the Teeth (r = 0.29), Diarrhea (r = 0.22), and Weight gain (r = -0.27) scale, the OHIP-14 correlated moderately to highly with the other QLQ-C30 and QLQ-H&N35 scales (Table 3).

**Table 2:**
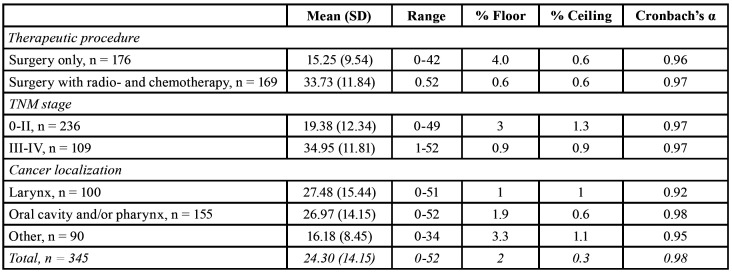
Main descriptive statistics of OHIP-14 scores.

**Table 3:**
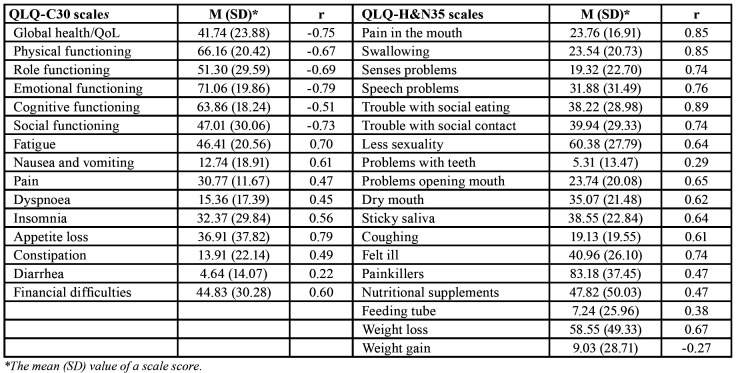
Correlations (r) of the OHIP-14 with the QLQ-C30 and QLQ-H&N35 scales.

## Discussion

The OHIP-14 was developed as short instrument to represent functional limitation, physical pain, psychological discomfort, physical, psychological and social disability, and overall handicap in relation to oral disorders (13). Available psychometric studies showed that its 14 items are best representing either one single underlying factor (15), three correlated factors (15,23), or seven correlated factors (13-16) of oral health. The results of our factor analyses indicate that all 14 items explain substantial proportion of the variance in oral health as one factor and this model was generally confirmed in the CFA, donating on the unidimensionality of the instrument. The results of the CFA showed that the model of three and seven correlated factors had minimally higher fit indexes in comparison to the one factor model, with the RMSEA remaining practically the same; marginal. In addition, sound internal consistency of the total score of 14 items, high Cronbach’s coefficients and high corrected item-total correlations as previously reported (13-23), indicate similar co-variances between the items and high homogeneity when used in a one single score. Thus, together our data for factor analyses and internal consistency indicate that the OHIP-14 for patients with HNC is best to be used as a unidimensional instrument, which is in line with previous data with other populations (15).

Further analyses showed that the OHIP-14 has appropriate aspects of construct validity, namely known-group and convergent/discriminant validity, what is also in line with the previous findings from studies with other populations (14,20,23). Based on the results of the t-test and ANOVA, OHIP-14 scores were likely linked to expected levels of compromised oral health due to the condition or therapeutic procedures, in such a way that an OHIP-14 score would be higher if oral health is more compromised and vice versa. In addition, the OHIP-14 correlated moderately to highly with the QLQ-C30 functioning and its theoretically similar symptom scales and the QLQ-H&N35 scales, indicating that the lower the functioning and higher the symptoms are, the greater the OHIP-14 score is (i.e., sound convergent validity). Lower correlations of the OHIP-14 with the scales Dyspnea, Constipation, Diarrhea, Pain killers, Nutritional supplements, Feeding tube, and Weight gain donate on sound discriminant validity. However, of particular importance are correlation with the QLQ-H&N35 scales Pain, Swallowing, Senses problems, Speech problems, Trouble with social eating, Trouble with social contact, Opening mouth, Dry mouth, and Sticky saliva, which were high, indicating on the overlapping of the measuring construct of the OHIP-14 with the measuring aspects of these scales. In this regard, the use of OHIP-14 could replace the QLQ-H&N35, at least the mentioned scales, in patients with HNC especially when it comes to measure oral health or oral QoL as one general indicator (i.e., one score).

There are some limitations to be acknowledged regarding the methodology of our study. It should be noted that only patients who agreed to participate were included and the sampling was convenient, thus those who may have less or more advanced cancer stages might not have participated. Additional psychometric aspects, such as screening properties, test-retest, responsiveness or predictive validity, and diagnostic value were also not tested.

Summarizing, the OHIP-14 is measuring a single underlying factor of oral health among patients with HNC, with sound internal consistency and construct validity. This study generalizes its use and opens the space for further use of OHIP-14 with these patients in research and clinical setting.
